# Discovery and fine-mapping of kidney function loci in first genome-wide association study in Africans

**DOI:** 10.1093/hmg/ddab088

**Published:** 2021-03-30

**Authors:** Segun Fatumo, Tinashe Chikowore, Robert Kalyesubula, Rebecca N Nsubuga, Gershim Asiki, Oyekanmi Nashiru, Janet Seeley, Amelia C Crampin, Dorothea Nitsch, Liam Smeeth, Pontiano Kaleebu, Stephen Burgess, Moffat Nyirenda, Nora Franceschini, Andrew P Morris, Laurie Tomlinson, Robert Newton

**Affiliations:** 1 Non-Communicable Disease Theme, MRC/UVRI and LSHTM, Entebbe, Uganda; 2 Department of Non-Communicable Disease Epidemiology, London School of Hygiene and Tropical Medicine London, London, UK; 3 H3Africa Bioinformatics Network (H3ABioNet) Node, Centre for Genomics Research and Innovation, NABDA/FMST, Abuja, Nigeria; 4 MRC/Wits Developmental Pathways for Health Research Unit, Department of Pediatrics, Faculty of Health Sciences, University of the Witwatersrand, Johannesburg, South Africa; 5 Sydney Brenner Institute for Molecular Bioscience, Faculty of Health Sciences, University of the Witwatersrand, Johannesburg, South Africa; 6 Departments of Physiology and Internal Medicine, Makerere University College of Health Sciences, Kampala, Uganda; 7 Department of Public Health and Primary Care & MRC Biostatistics Unit, University of Cambridge, UK; 8 Department of Epidemiology, University of North Carolina, Chapel Hill, NC, USA; 9 Health and Systems for Health Research Unit, African Population and Health Research Center, Nairobi, Kenya; 10 Centre for Genetics and Genomics Versus Arthritis, Centre for Musculoskeletal Research, Division of Musculoskeletal and Dermatological Sciences, The University of Manchester, Manchester, UK

## Abstract

Genome-wide association studies (GWAS) of kidney function have uncovered hundreds of loci, primarily in populations of European ancestry. We have undertaken the first continental African GWAS of estimated glomerular filtration rate (eGFR), a measure of kidney function used to define chronic kidney disease (CKD). We conducted GWAS of eGFR in 3288 East Africans from the Uganda General Population Cohort (GPC) and replicated in 8224 African Americans from the Women’s Health Initiative. Loci attaining genome-wide significant evidence for association (*P* < 5 × 10^−8^) were followed up with Bayesian fine-mapping to localize potential causal variants. The predictive power of a genetic risk score (GRS) constructed from previously reported trans-ancestry eGFR lead single nucleotide polymorphism (SNPs) was evaluated in the Uganda GPC. We identified and validated two eGFR loci. At the glycine amidinotransferase (*GATM*) locus, the association signal (lead SNP rs2433603, *P* = 1.0 × 10^−8^) in the Uganda GPC GWAS was distinct from previously reported signals at this locus. At the haemoglobin beta (*HBB*) locus, the association signal (lead SNP rs141845179, *P* = 3.0 × 10^−8^) has been previously reported. The lead SNP at the *HBB* locus accounted for 88% of the posterior probability of causality after fine-mapping, but did not colocalise with kidney expression quantitative trait loci. The trans-ancestry GRS of eGFR was not significantly predictive into the Ugandan population. In the first GWAS of eGFR in continental Africa, we validated two previously reported loci at *GATM* and *HBB*. At the *GATM* locus, the association signal was distinct from that previously reported. These results demonstrate the value of performing GWAS in continental Africans, providing a rich genomic resource to larger consortia for further discovery and fine-mapping. The study emphasizes that additional large-scale efforts in Africa are warranted to gain further insight into the genetic architecture of CKD.

## Introduction

Chronic kidney disease (CKD) is a global public health problem, with adverse outcomes of kidney failure, cardiovascular disease and premature death. CKD is at least three times more frequent in Africa, which has limited resources, than in developed countries (1). With rapidly increasing urbanization, trends towards unhealthy diets, obesity and increases in metabolic risk factors, the projected increase in the prevalence of CKD may be even greater in Africa compared with developed countries (2). The interplay of genomic and environmental factors contributes to this complex heterogeneous disease. However, CKD heritability is estimated to be as high as 30–75% (3). Genetic variants associated with CKD may be population specific, indeed the association of the *APOL1* locus with CKD, which has risen to high frequencies within West Africa due to selection pressures related to protection against Lassa fever, highlights the potential for novel discovery in African populations (3). Given the fundamental significance of Africa to our human origins, there is a strong scientific need to establish large-scale efforts examining the genetic contribution to disease susceptibility across diverse populations within Africa (2,5,6). The clear genomic diversity and allelic differentiation among various African populations, in addition to the lower linkage disequilibrium (LD) between genetic variants, provides an outstanding opportunity to gain new insights into disease aetiology and genetic fine-mapping that have relevance for all ancestry groups (9,10). However, despite the value of conducting such research in Africa, there is no known genome-wide association study (GWAS) of kidney function in continental Africa, with published studies of African ancestry individuals being limited to African Americans (9,10). Whilst African Americans typically have a large proportion of West African ancestry, several studies have shown that the genetic architecture of African Americans is distinct from that of Africans from continental Africa (7). The African American population reflects admixture of people of West and Central-West African descent, adding to the relevance of studying populations from other regions of Africa.

Here, we conducted the first continental African GWAS of estimated glomerular filtration rate (eGFR), a measure of kidney function used to define CKD, including 3288 individuals from the Uganda General Population Cohort (GPC). Subsequently, associations were validated through GWAS of eGFR in a large sample of 8224 African Americans from the Women’s Health Initiative (WHI). Together, these GWAS comprised a total of 11 512 African ancestry individuals. We used the Uganda GPC to: (i) identify loci associated with eGFR; (ii) fine-map loci by taking advantage of the finer-grained LD structure in African ancestry populations and (iii) evaluate the predictive power of an eGFR genetic risk score (GRS) into the Ugandan population that was derived from lead single nucleotide polymorphism (SNPs) at previously reported loci from trans-ancestry GWAS meta-analysis (9,10).

## Results

### Discovery genetic association

**Figure 1:**
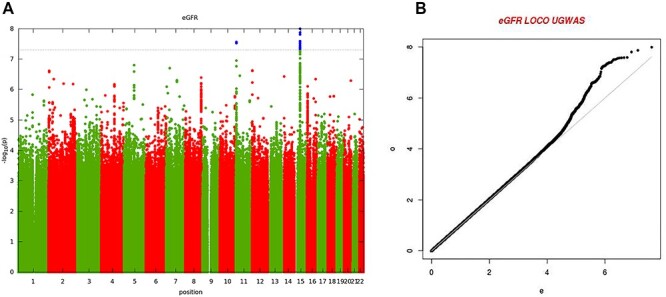
(**A**) Manhattan plot of genome-wide associations of eGFR in 3288 Ugandan individuals from the GPC. Each point denotes a variant with MAF > 0.5%, with the *x*-axis representing the genomic position and *y*-axis representing the strength of association (−log_10_  *P*-value). The dotted line shows the genome-wide significance threshold of *P* < 5 × 10^−8^. (**B**) Quantile-Quantile plot of genome-wide associations of eGFR: the genome-wide genomic control inflation factor was 1.01.

**Figure 2:**
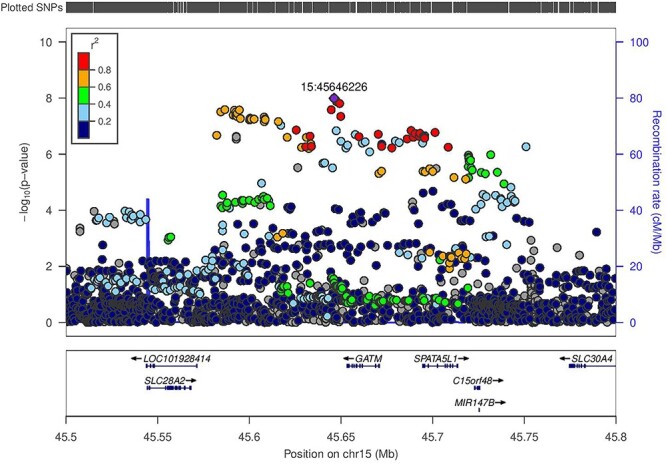
Regional association plot for eGFR in 3288 Ugandan individuals from the GPC at the *GATM* locus. The lead SNP rs2433603 (15:45646226) (*P* = 1.0 × 10^−8^) is coloured in purple. LD (*r*^2^) with other SNPs at the locus was calculated on the basis of the Ugandan SNP genotypes used in this study.

**Figure 3:**
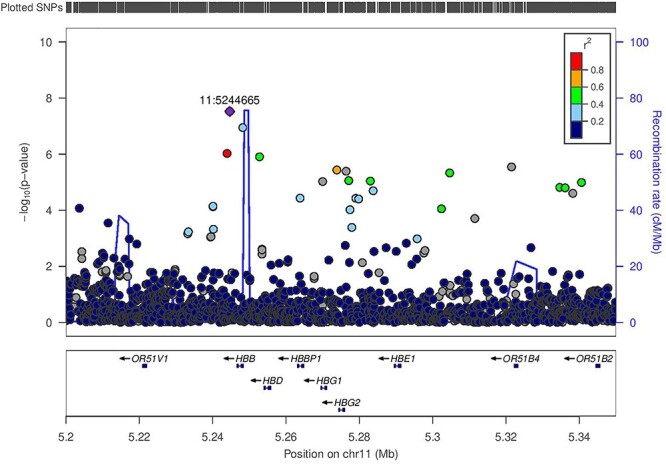
Regional association plot for eGFR in 3288 Ugandan individuals from the GPC at the *HBB* locus. The lead SNP rs141845179 (11:5244665) (*P* = 3.0 × 10^−8^) is coloured in purple. LD (*r*^2^) with other SNPs at the locus was calculated on the basis of the Ugandan SNP genotypes used in this study.

The characteristics, quality control and imputation of the 3288 Uganda GPC study participants are shown in the Methods. We analyzed associations of eGFR for 20 594 556 SNPs that met an minor allele frequency (MAF) threshold of at least 0.5% in a merged panel of imputed GWAS and whole-genome sequences. We tested for association in a linear mixed model implemented in genome-wide efficient mixed-model association (GEMMA), which accounted well for population structure and relatedness. Our association analysis in the Uganda GPC showed no evidence of residual population structure with a genomic inflation factor (*λ*) of 1.01. We identified two loci attaining genome-wide significance (*P* < 5 × 10^−8^) in GPC (Table 1, Fig. 1) mapping to glycine amidinotransferase (*GATM*) (lead SNP rs2433603, MAF = 48%, *P* = 1.0 × 10^−8^) (Fig. 2) and haemoglobin beta (*HBB*) (lead SNP rs141845179, MAF = 8%, *P* = 3.0 × 10^−8^) (Fig. 3). Both loci have been previously reported as associated with eGFR in European ancestry and trans-ancestry GWAS meta-analyses (10,11).

**Table 1:** Loci attaining genome-wide significant (*P* < 5 × 10^−^8) association with eGFR after meta-analysis of GPC and WHI in up to 11 512 individuals of African ancestry

						Uganda				WHI				Meta-Analysis	
Locus	Lead SNP	Chr	BP (b37)	EA	NEA	Beta	SE	MAF	*P*-value	Beta	SE	*P*-value	MAF	*P*-value	*N*
*GATM*	rs2433603	15	45 646 226	T	C	0.145	0.025	48%	1.0 × 10^−8^	1.0276	0.3042	7.3 × 10^−4^	47%	2.4 × 10^−9^	11 512
*HBB*	rs141845179	11	5 244 665	G	T	0.266	0.048	8%	3.0 × 10^−8^	NA	NA	NA	NA	3.0 × 10^−8^	3288

SNP, single nucleotide polymorphism; Chr, Chromosome; BP, base position; EA, effect allele; NEA, Non-effect allele; SE, standard error; NA, not available.

To investigate the relationships between the association signals identified in the Uganda GPC and those reported in other populations, we performed conditional analyses. The identified genetic variant at *GATM*, rs2433603, was distinct from previously reported associations (rs1145077, rs1153855 and rs1145093) at this locus (conditional *P* = 4.0 × 10^−7^). This genetic variant is monomorphic in European ancestry populations and rare in East Asian ancestry populations in the 1000 Genomes Project Phase 3 (12). This variant has MAF of 48% in Uganda GPC, and 44% and 37%, respectively, in Luhya in Webuye (LWK), Kenya and Yoruba in Ibadan (YRI), Nigeria in the 1000 Genomes Project Phase 3 (12). At the *HBB* locus, after conditioning on the previously reported SNP (rs334) at this locus, the association with the lead SNP (rs141845179) was no longer significant (conditional *P* = 0.024). This is because the lead SNP (rs141845179) is in strong LD (*r*^2^ = 0.95 in Uganda GPC) with the sickle cell SNP (rs334), and therefore, they reflect the same signal. Of note, the lead SNP has MAF of 8% in Uganda but is much less frequent in admixed Africans in the 1000 Genomes Project Phase 3 (1% in African Americans from Southwest USA and 2% in Afro-Carribeans from Barbados). The variant (rs334) in *HBB* has also been previously associated with other kidney traits, including urinary albumin to creatinine ratio and CKD in both African Americans and US Hispanics/Latinos (13,14).

To investigate the association of previously reported eGFR loci in Uganda, we also conducted a look-up of 308 lead SNPs from the largest published meta-analysis of eGFR (10) (Supplementary Material, Table S1). Of these, 281 variants were also reported in the GPC GWAS. We observed an enrichment of SNPs with nominal evidence of association (*P* < 0.05) in GPC and with the same direction of effect as in the previously reported eGFR meta-analysis (22 SNPs observed, 7.0 expected, binomial test *P* = 3.2 × 10^−6^). We also replicated eGFR associations of important African population specific variants near *APOL1/MYH9* (15) (Supplementary Material, Table S2).

The G2 allele is tagged by the deletion rs71785313, which is monomorphic in the Uganda GPC. The G1 allele is tagged by two SNPs: rs73885319 and rs60910145. There was no person homozygous for the G risk allele for rs60910145 and as such we could not fit the recessive model for this SNP. For rs73885319, there were 17 carriers of the homozygous risk genotype, but there was no evidence of association with eGFR in the Uganda GPC (*P* = 0.22) (Supplementary Material, Table S3). This lack of association could reflect low power given the low sample size.

### Replication of eGFR associations in WHI

Lead SNPs showing strong evidence of association (*P* < 5 × 10^−5^) in Uganda were considered for replication and meta-analysis in WHI (Supplementary Material, Table S4). We replicated the association signal at the lead SNP at the *GATM* locus in WHI (*P* = 0.00073, meta-analysis *P* = 2.4 × 10^−9^). The lead SNP at the *HBB* locus was not available in WHI, and it is rare or monomorphic in other populations. However, as described above, rs334 is a close proxy for the lead SNP at the *HBB* locus and represents the same eGFR signal. This SNP was available in WHI, where the association was replicated (*P* = 0.017, meta-analysis *P* = 1.2 × 10^−6^). No other proxies for the lead SNP at the *HBB* locus (*r*^2^ > 0.9 in the Uganda GPC) were associated with eGFR in WHI (Supplementary Material, Table S5). No other loci attained genome-wide significant evidence of eGFR association after meta-analysis of the Uganda GPC and WHI.

### Fine-mapping of loci attaining genome-wide significance

Bayesian fine-mapping of the *GATM* and *HBB* loci was undertaken in the region mapping 500 kb up- and down-stream of each lead SNP, on the basis of association summary statistics from the meta-analysis of GPC and WHI. At the *GATM* locus, the 99% credible set consisted of 63 variants, and no variant accounted for >50% of the posterior probability (the lead SNP, rs1145092 had a posterior probability of 13%) (Supplementary Material, Table S6). At the *HBB* locus, the 99% credible set consisted of 73 variants, but with the lead SNP, rs141845179, accounting for 88% of the posterior probability (Supplementary Material, Table S7).

### Colocalization of eGFR association signals with expression quantitative trait locus (eQTLs)

To gain insight into the causal genes through which eGFR association signals at the *HBB* and *GATM* loci are mediated, we first considered kidney eQTL in Genotype-Tissue Expression (GTEx) (16), NephQTL (17), the Human Kidney eQTL Atlas (18) and RegulomeDB (19). The lead SNPs were not significant eQTLs in kidney tissue in any of these resources. The lead SNP at the *GATM* locus, rs2433603, is a significant eQTL for three genes in multiple non-kidney tissues in GTEx (Supplementary Material, Table S8). However, rs2433603 is not in strong LD with the respective lead eQTL SNPs, and we cannot therefore conclude that the eGFR association and eQTLs colocalize. The lead SNP at the *HBB* locus, rs141845179, is not a significant eQTL for any gene/tissue in GTEx. We extended our investigations to other variants in the 99% credible set for each locus and interrogated their regulatory impact using RegulomeDB (Supplementary Material, Tables S9 and S10). Two variants at the *GATM* locus (rs2668747 and rs1153850) have support for regulatory impact from eQTL data and transcription factor binding. These SNPs showed the strongest associations with expression of *SPATA5L1* in cultured fibroblasts and whole blood in GTEx but were not in strong LD with the lead eQTL SNP (rs1365610; *r*^2^ = 0.076 with rs2668747; *r*^2^ = 0.47 with rs1153850) and thus did not support colocalization with the eGFR signal.

### Transferability of trans-ancestry eGFR GRS into Uganda

We used lead SNPs from trans-ancestry a meta-analysis (9) of eGFR to evaluate the predictive power of an unweighted GRS into unrelated individuals in the Uganda population (Table 2). We were unable to undertake a weighted GRS because different transformations of the trait were performed in the trans-ancestry meta-analysis and in the GPC GWAS. Because the SNP effects were aligned to the eGFR decreasing allele, we expected an increased score to be associated with lower eGFR. Whilst the GRS showed the correct direction of effect, it was not significantly associated with eGFR in the Uganda population (*P* = 0.076) and accounted for only 0.04% of the trait variance after accounting for age, sex and principal components to adjust for population structure. We also leverage the largest trans-ancestry meta-analysis of eGFR (10) to assess the predictive power of a weighted GRS into unrelated individuals in the Uganda population. This GRS also showed the correct direction of effect though not significantly associated with eGFR in the Uganda population (*P* = 0.524) and accounted for only 0.01% of the trait variance after accounting for age, sex and principal components to adjust for population structure (Table 3).

**Table 2:** Regression coefficients for the association of the GRS with eGFR in the Ugandan population

Variable	Model without GRS			Full model		
	Beta (SE)	*P*	*R* ^2^	Beta (SE)	*P*	*R* ^2^
Age	−0.92 (0.01)	2.0 × 10^−16^	0.5492	0.93 (0.015)	2.0 × 10^−16^	0.5496
Sex	−2.53 (0.56)	7.9 × 10^−6^		−2.55 (0.53)	6.8 × 10^−6^	
PC1	22.62 (21.53)	0.294		21.77 (21.53)	0.312	
PC2	−40.46 (22.01)	0.066		−41.78 (22.01)	0.058	
PC3	32.57 (20.17)	0.106		33.18 (20.16)	0.099	
PC4	24.34 (23.62)	0.303		24.62 (23.61)	0.297	
PC5	−2.95 (21.05)	0.889		−3.40 (21.04)	0.872	
GRS				−0.17 (0.09)	0.076	

**Table 3:** Regression coefficients for the association of weighted GRS derived from Wuttke *et al*. (10) with eGFR in the Ugandan population

Variable	Model without GRS			Full model		
	Beta (SE)	*P*	*R* ^2^	Beta (SE)	*P*	*R* ^2^
Age	−0.92 (0.01)	2.0 × 10^−16^	0.5513	−0.92 (0.01)	2.0 × 10^−16^	0.5512
Sex	−2.45 (0.56)	1.49 × 10^−5^		−2.44 (0.56)	1.53 × 10^−5^	
PC1	42.86 (14.81)	0.294		42.88 (14.81)	0.003	
PC2	−11.08 (14.82)	0.454		−10.60 (14.84)	0.475	
PC3	42.23 (14.80)	0.004		42.42 (14.81)	0.004	
PC4	−9.59 (14.81)	0.517		−10.17 (14..83)	0.492	
PC5	29.97 (14.82)	0.043		30.02 (14.82)	0.043	
GRS				−12.38 (19.43)	0.524	

### Sex-stratified analysis of lead SNPs in Uganda GPC

We performed a stratified analysis by sex in the Uganda samples to determine if any heterogeneity between male and female in order to ascertain if this contribute to lack of replication in WHI dataset being comprised of only women. Our analysis shows no heterogeneity (Supplementary Material, Table S11).

## Discussion

In the first GWAS of eGFR in continental Africa, we validated previously reported eGFR loci mapping to *GATM* and *HBB*. The association in Ugandans at the *GATM* locus is driven by an African-specific variant (MAF of 48% in Uganda GPC) and distinct from those previously reported SNP in European ancestry and trans-ancestry GWAS (9,10). The lead SNP at the *HBB* locus was in strong LD with the previously reported sickle cell missense variant rs334 (11) and represented the same underlying eGFR signal. *GATM* encodes a mitochondrial enzyme that belongs to the amidinotransferase family. This enzyme is involved in creatinine biosynthesis, whereby it catalyzes the transfer of a guanido group from L-arginine to glycine, resulting in guanidinoacetic acid, the immediate precursor of creatinine. The *HBB* gene provides instructions for making a protein called beta-globin. Beta globin protein changes related to rs334 causes sickle cell anaemia. Absence of beta chain causes beta-zero-thalassemia, and reduced amounts of detectable beta globin cause beta-plus-thalassemia.

Bayesian fine-mapping revealed that the lead SNP, rs141845179, accounted for 88% of the posterior probability of driving the association signal at the *HBB* locus. At the GATM locus, the association signal was less precisely refined, with the lead SNP, rs2433603, accounting for just 13% of the posterior probability. Unfortunately, the lead SNPs were not significant eQTLs in kidney tissue in publicly available kidney gene expression resources. The lack of colocalization could reflect the fact that these variants are rare in European ancestry populations and are thus not well represented in the European-centric eQTL resources used in this investigation, reemphasizing the need for well powered eQTL studies in Africans. Approaches such as coloc (36) compare patterns of association between the trait and expression and therefore depend on LD. We were concerned that the differences on LD structure between the populations contributing to Uganda GPC (African ancestry) and the expression resources (European ancestry) would invalidate the colocalization. *GATM* protein is a renal proximal tubular enzyme involved in the creatinine biosynthetic pathway, and recent studies have shown that fully penetrant heterozygous mutations in the *GATM* gene lead to intra-mitochondrial fibrillary deposition and clinical manifestations of Fanconi syndrome and CKD (20,21). It is possible that the signal at the *GATM* locus reflects associations with the biomarker used to estimate kidney function (serum creatinine), but genetic studies in populations are not suited to address this. Many serum creatinine-based eGFR loci have not been associated with CKD at genome-wide significance. Whilst this may reflect lower power to detect association with the disease outcome, a subset of these loci may represent true genetic influences on physiologic variation of eGFR but not CKD risk. The GRS derived from previously reported trans-ancestry lead SNPs (11) for eGFR was significantly predictive of eGFR in the Ugandan population. These results show that there is a shared genetic contribution to eGFR at established loci in continental Africans compared with African Americans.

The GRS derived from previously reported trans-ancestry lead SNPs for eGFR was not significantly predictive in the Ugandan population. This is mostly likely due to a lack of power in GPC because of small sample size. However, the lack of transferability could also be because the way eGFR is calculated in continental Africa. Studies have shown that there is potential error measurement of serum creatinine in continental Africa that might lead to inaccurate estimates of kidney disease at individual and population level (22). To address this issue, a Laboratory Working Group of the National Kidney Disease Education Programme published recommendations in 2006 to standardize how the creatinine is measured (22). In this study, eGFR was calculated using the CKD-Epi formula, without use of the coefficient for African Americans (22,23). The absence of a validated estimating equation for kidney function in Africans could be a contributing factor to the lack of GRS transferability. Another potential explanation for why the GRS was not significant is because the lead SNPs from the trans-ancestry analysis might not themselves be causal variants and are not in LD with the causal variant in the Ugandan population because of differences in LD structure between ancestry groups. The trans-ethnic meta-analyses include only variants that are common across populations and therefore disfavour some important population-specific variants such as *APOL1* and *HBB* variants.

We attempted to replicate signals from the largest previous GWAS meta-analysis of eGFR, which included individuals predominantly of mostly European ancestry (10). After correction for multiple testing, none of the previously reported lead SNPs from that study showed significant evidence of association with eGFR in the Uganda GPC. There are several reasons why this could occur. First, the previously reported lead SNP might not be causal and is a poor tag for the causal variant in the Ugandan population. Second, the causal variant could be very rare or monomorphic in the Ugandan population, and therefore, an association would not be detected. Third, the African Americans used in the replication set are predominantly West African ancestry individuals, which might have limited replication of the Uganda GPC of East African ancestry. Finally, because the effect sizes are small, there will be limited power to detect association with the sample size available in the Uganda GPC. However, we note that there was an enrichment of signals with the same direction of effect from the largest published meta-analysis of eGFR (10) and the Uganda GPC, which suggests shared underlying causal variants that could be identified with larger sample sizes and/or by testing a better tag for the causal variant in the Ugandan population.

Despite the relatively high burden of CKD in Africa (29), there have been no previous GWAS of eGFR in continental Africa. One limitation of this first GWAS is that, with a small sample size, we are underpowered to reliably detect associations at genome-wide significance thresholds. We have applied the traditional threshold of genome-wide significance (p < 5x10^−8^), which was originally defined on the basis of LD structure in European ancestry populations. A more stringent threshold of *P* < 5 × 10^−9^ has been proposed in African ancestry populations, to account for shorter range LD than in those of European ancestry (8). After meta-analysis, the novel association signal at the *GATM* locus attains this stringent threshold. Whilst the association signal at *HBB* does not, it has been reported in previous in GWAS (11), and thus can be considered confirmation of previous results in a new population, rather than novel discovery.

Our findings further highlight the importance of diverse ancestries for uncovering novel associations. Larger continental African meta-analyses are warranted to gain further insight on the genetic architecture of eGFR. In addition, although GWAS still remains a leading tool to identify loci contributing to complex diseases, to follow up significant findings and gain biological insights, the multi-omics resources that would inform these analyses need to be better represented in Africans. The study of populations in Africa provides a research framework to help characterize ethnic-specific patterns of variation in CKD among populations (25) and in a larger framework of studies might also help identify population-specific genetic or environmental factors that may statistically interact with identified genetic loci. Given these scientific opportunities, the ascertainment and collation of genetic epidemiological resources with the statistical resolution to examine these associations in African populations is a high priority.

## Materials and Methods

### GPC study participants

The recruited African individuals were part of the nine ethno-linguistic groups from the Uganda GPC. GPC is a population-based cohort of roughly 22 000 inhabitants around 25 neighbouring villages of Kyamulibwa, which is a subcounty of Kalungu district in the countryside in the south-west of Uganda. The cohort study was founded in the late 1980s by the Medical Research Council (MRC) UK in partnership with the Uganda Virus Research Institute (UVRI) to primarily investigate the trends in incidence and prevalence of HIV infection in Uganda. Samples were collected from research participants during a survey from the research study area. The study area is clustered into villages defined by governmental borders ranging in size from 300 to 1500 dwellers and includes numerous families who are resident within households (26). The GPC Round 22 study took place in 2011 through collaboration between the University of Cambridge, Wellcome Sanger Institute and MRC/UVRI in Uganda. This study was approved by the Science and Ethics Committee of the UVRI, the Ugandan National Council for Science and Technology and the East of England-Cambridge South NHS Research Ethics Committee United Kingdom. The study was contained within one annual survey round of the longitudinal cohort. The focus of the GPC Round 22 study was to investigate the genetics and epidemiology of communicable and non-communicable diseases to provide aetiological insights into the genetic variation in communicable and non-communicable diseases.

### GPC study design

The data collection of GPC Round 22 study consisted of five main stages, which took place in 2011 over the course of the year: mobilization (recruitment and consenting), mapping, census, survey and feedback of results and clinical follow-up. The census consisted of a family questionnaire and questionnaire for the individual recruited from within the family. The family questionnaire was completed by the head of family or another responsible adult or emancipated minor member of the household. The household census questionnaire focused on sociodemographic information about the household, such as the quality of the house, property ownership and employment of workers. The individual survey questionnaire captured information on members of a household including position within household, marital status, resident status, childbirth and fertility, tribe and religion. Information on lifestyle and health was obtained using a standard questionnaire. This included biophysical measurements and blood samples (26). We genotyped 5000 and sequenced 2000 samples from nine ethno-linguistic groups from the GPC, which includes related individuals.

### GPC genotyping and quality control

Individuals (*n* = 5000) were genotyped on the Illumina HumanOmni2.5-8 array, and 4872 were retained following a pre-quality control stage. GWAS genotype data were subjected to stringent quality control filtering. Of a total of 2 314 174 autosomal variants genotyped, 39 368 were excluded because they did not pass SNP quality thresholds for call rate (<97%, *n* = 25 037 SNPs) and deviation from Hardy–Weinberg equilibrium (HWE) (*P* < 10^−8^, *n* = 14 331 SNPs) as reported in (8). We excluded 91 individuals who failed to meet the quality control for call rate (>97%) or had gender mismatch compared with X-chromosome. We carried out further quality control for the GWAS analysis, for which three samples were excluded as heterozygosity outliers (heterozygosity ≥3 SD from mean). Additional six samples were excluded due to potential contamination.

### Curation of GPC sequence data

An additional 2000 Uganda samples (UG2G) underwent low coverage whole-genome sequencing on the Illumina HiSeq 2000 with 75 bp paired end reads, at low coverage, with an average coverage of 4× for each sample. In total, 1978 of them passed quality control. The workflow for data processing and description of UG2G has been previously described in more detail (5,6). Briefly, after the generation of raw reads on Illumina HiSeq sequencing machine, the reads were converted to BAM format using Illumina2BAM. We Mapped the Human samples using the BWA backtrack algorithm with the GRCh37 1000 Genomes phase II reference.

### GPC haplotype phasing and imputation into genotype data

Haplotype phasing of GWAS data was carried out using SHAPEIT2 (27) with standard parameters. A previous study has shown that phasing with SHAPEIT2 in this cohort with dense genotype data provides very high accuracy even when pedigree structure is not explicitly specified during phasing (28).

Imputation of the pre-phased genotype data was carried out with IMPUTE2 (29) using a merged reference panel of the whole-genome sequence data from the African Genome Variation Project (30), the UG2G described earlier and the 1000 Genomes Project phase 3 (1000 Genomes Project Consortium, 2015) (12) following standard recommendations. Imputation was carried out in chunks of 2 MB and then concatenated. Imputed SNPs were further filtered for info quality >0.3 and an MAF >0.5%. ‘Duplicate variants were removed post imputation. We removed both duplicates as we did not consider this to be reliable.’

### Merging of GPC genotype and sequence data

The final dataset used for this analysis included merged genotype data on 4772 and sequence data on 1978 individuals. We note that there are 343 individuals who have been genotyped and sequenced; for these individuals, we only included the sequence data and not the genotype data. The final dataset, therefore, included 6407 individuals (4429 with genotype and imputed data and 1978 with sequence data).

Following merging, we assessed and removed any systematic differences between imputed genotype data and sequence data. We did this by carrying out principal component analysis using merged data for the 343 individuals who had been genotyped and sequenced in duplicate to examine whether there was separation by data mode (imputed genotype data and sequenced data). Full details were reported in (5). For GWAS analyses, we only included a subset of variants (*n* = 20 594 556) that met an MAF threshold of at least 0.5%.

### GPC laboratory test and phenotype definition

Creatinine was measured using the enzymatic method traceable to an isotope dilution mass spectrometry method (IDSM) (31). Collectively, the serum creatinine level was measured in 3288 Uganda individuals for Round 22 (23). The eGFR was calculated using the CKD-Epi formula, without use of the coefficient for African Americans (22). We carried out the inverse rank normal transformation of eGFR residuals after adjusting for age, age^2^ and sex.

### Statistical methods for association analysis in GPC

GWAS was performed using the standard mixed-model approach implemented in GEMMA version 24 (32) for analysis of pooled data from 3288 individuals (2266 genotyped and 1022 whole genome sequenced individuals have eGFR measurements) in GPC and tested association of eGFR, under an additive model, with 20 594 556 SNPs that met an MAF threshold of at least 0.5% in the merged panel of imputed GWAS and whole-genome sequences. To maximize discovery, we used the leave one chromosome out (LOCO) approach for analysis (5,6). In this approach each chromosome is excluded from generation of the kinship matrix in turn, for association analysis for markers along that chromosome. This ensures that causal SNPs at a locus on a given chromosome are not used for generation of the kinship matrix used in analysis of that specific chromosome. Therefore, we generated 22 kinship matrices for analysis, each excluding the chromosome being analyzed using the given matrix. For computational efficiency, and to avoid correlation effects due to LD, we LD pruned the data prior to calculation of the kinship matrix for each LOCO analysis.

For all loci attaining genome-wide significance that have been previously reported in GWAS of eGFR, we performed conditional analyses in GEMMA to determine whether the association signals were distinct. Specifically, we included genotypes under an additive model at previously reported lead SNPs as a fixed effect in the mixed model. We also searched for evidence of multiple distinct signals of association in GPC by including genotypes at the lead SNP as a fixed effect in the mixed model.

### WHI study design

The WHI is a study of postmenopausal women and health outcomes, funded by the National Heart Lung and Blood Institute. A total of 161 808 women aged 50–79 years old were recruited from 40 clinical centres in the United States between 1993 and 1998. Study protocols and consent forms were approved by the institutional review boards at all participating institutions. The WHI SHARe minority cohort includes 8515 self-identified African American women, who provided written informed consent for study participation and DNA analysis.

### WHI genotyping, imputation and phenotype transformation

African American women who consented to genome-wide scanning underwent genotyping with the Affymetrix Genome-Wide HumanSNP Array 6.0 containing 906 000 SNPs. The samples underwent initial quality control including removal of samples with poor DNA quality, abnormal sex chromosomes, relatedness and low call rates as previously reported (9). Additional quality control measurements were made at the SNP level assessing for HWE (goodness-of-fit χ2 > 10), call rates 90%, monomorphic SNPs and MAFs 1%. We used frappe to estimate individual admixture, and estimates were included in models to account for population stratification.

After quality control, GWAS scaffolds were pre-phased and imputed using MaCH/minimac *r*^2^ ≥ 0.3, and 13 096 173 SNVs passing quality control were tested for association with eGFR. For each individual, eGFR was calculated from serum creatinine (mg/dl, IDSM measured assay) using the Modification of Diet in Renal Disease equation.

### Replication and meta-analysis

All lead SNPs (separated by 500 kb) apart showing strong evidence of association (*P* < 5 × 10^−5^) in the Uganda GPC were considered for replication in WHI. In view of the different scales of the effect sizes, association summary statistics of Uganda GPC and WHI were aggregated using the fixed-effects meta-analysis on the basis of the sample size weighting of *Z*-scores (Stouffer’s method) in METAL (33).

### Sex-stratified analysis

In order to ascertain if female-only replication cohort might limit our finding, we stratified the Uganda GPC dataset by sex. eGFR association testing was performed separately in males and females using GEMMA version 24, and the results were combined scores Stouffer’s method implemented in METAL (33).

### Recessive model of *APOL1* G1/G2 risk allele

*APOL1* G1/G2 alleles have been reported to be strongly associated with kidney disease in individuals of African ancestry. We attempted to fit the recessive model for the two *APOL1* risk haplotypes (G1, tagged by rs73885319 A > G and rs60910145 T > G; G2, tagged by rs71785313 TTATAA/−). This was done by recoding the genotypes of each SNP for carriage of the homozygous risk genotype. We tested for association with eGFR under this recessive model via linear regression whilst correcting for age, sex and principal components as covariates using only unrelated individuals from the Uganda GPC.

### Fine-mapping

To fine-map the *GATM* and *HBB* loci, we first conducted meta-analysis (as described above) of association summary statistics from Uganda GPC and WHI for all SNPs mapping within 500 kb of the lead SNP at each locus. We used a Bayesian approach (34) to fine-map the two loci, where the meta-analysis *Z*-score for the *i*th SNP, denoted *Z_i_*, was used to compute a Bayes’ factor in favour of association, denoted ${BF}_i$, given by

$${BF}_i={e}^{\left[\frac{Z_i^2-\log (K)}{2}\right]},$$

where *K* is the number of studies. The posterior probability of driving the association for the *i*th SNP was then computed by

$${\pi}_i=\frac{BF_i}{\sum_jB{F}_j}$$

where the summation in the denominator is over all SNPs at the locus. Ninety-nine percent credible sets for each locus were derived by sorting the Bayes’ factors of the SNPs from the highest to the lowest, and then included SNPs needed to attain a cumulative posterior probability that is ≥0.99.

### eQTL analysis

We considered publicly available kidney eQTL resources from the GTEx Project (16), NephQTL (17), the Human Kidney eQTL Atlas (18) and RegulomeDB (19). We conducted a look up of the lead SNP at each locus for association with gene expression in kidney tissue using a range of publicly available resources: (i) kidney cortex in GTEx; (ii) glomerular and tubulointerstitial kidney tissue in NephQTL; (iii) whole kidney, glomerulus and tubules in the Human Kidney eQTL Atlas. As secondary analyses, we also conducted a look up of the lead SNP at each locus for association with gene expression in other tissues available in GTEx. Where the lead SNP was a significant eQTL in a given tissue, we assessed the extent of LD with the lead eQTL SNP in the Uganda GPC to evaluate the evidence in favour of colocalization. We also interrogated variants in the 99% credible set for evidence of regulatory impact using RegulomeDB.

### Genetic risk score

After removing 817 first-degree relatives from the Uganda GPC cohort derived from PIHAT values > 0.5, we calculated principal components using –pca in PLINK (35). Lead SNPs with effects on trait aligned to the eGFR decreasing allele from a previously published trans-ancestry meta-analysis (9) of eGFR (*n* = 120 SNPs) and (*n* = 246 matching SNPs from (10)) were selected separately and used to compute an unweighted GRS by counting the number of eGFR decreasing alleles, using the allelic scoring approach in PLINK (35). The predictive power of the GRS was evaluated by assessing the change in *R*^2^ (variance explained) when it was added to the linear model of eGFR adjusted for age, sex and principal components.

## Supplementary Material

SupplementaryTables1to11a_ddab088

## Data Availability

All individual level data, phenotype, genotype and sequence data are available to researchers under managed access on EGA EGAS00001001558/EGAD00010000965. Requests for access to data will be granted for all research consistent with the consent provided by participants.
